# Mechanism for Recognition of an Unusual Mycobacterial Glycolipid by the Macrophage Receptor Mincle*[Fn FN2]

**DOI:** 10.1074/jbc.M113.497149

**Published:** 2013-08-19

**Authors:** Hadar Feinberg, Sabine A. F. Jégouzo, Thomas J. W. Rowntree, Yue Guan, Matthew A. Brash, Maureen E. Taylor, William I. Weis, Kurt Drickamer

**Affiliations:** From the ‡ Departments of Structural Biology and Molecular and Cellular Physiology, Stanford University School of Medicine, Stanford, California 94305 and; the § Department of Life Sciences, Imperial College, London SW7 2AZ, United Kingdom

**Keywords:** Carbohydrate-binding Protein, Crystal Structure, Glycobiology, Glycolipids, *Mycobacterium tuberculosis*, CLEC4E, Cord Factor

## Abstract

Binding of the macrophage lectin mincle to trehalose dimycolate, a key glycolipid virulence factor on the surface of *Mycobacterium tuberculosis* and *Mycobacterium bovis*, initiates responses that can lead both to toxicity and to protection of these pathogens from destruction. Crystallographic structural analysis, site-directed mutagenesis, and binding studies with glycolipid mimics have been used to define an extended binding site in the C-type carbohydrate recognition domain (CRD) of bovine mincle that encompasses both the headgroup and a portion of the attached acyl chains. One glucose residue of the trehalose Glcα1–1Glcα headgroup is liganded to a Ca^2+^ in a manner common to many C-type CRDs, whereas the second glucose residue is accommodated in a novel secondary binding site. The additional contacts in the secondary site lead to a 36-fold higher affinity for trehalose compared with glucose. An adjacent hydrophobic groove, not seen in other C-type CRDs, provides a docking site for one of the acyl chains attached to the trehalose, which can be targeted with small molecule analogs of trehalose dimycolate that bind with 52-fold higher affinity than trehalose. The data demonstrate how mincle bridges between the surfaces of the macrophage and the mycobacterium and suggest the possibility of disrupting this interaction. In addition, the results may provide a basis for design of adjuvants that mimic the ability of mycobacteria to stimulate a response to immunization that can be employed in vaccine development.

## Teaser

**Background:** Mincle facilitates establishment of persistent infections of macrophages by *Mycobacterium tuberculosis*.

**Results:** The mechanism of mincle binding to mycobacterial glycolipids has been defined, and inhibitors have been synthesized.

**Conclusion:** Mincle binds to both the sugar portion of the glycolipid and the hydrocarbon tail.

**Significance:** The results suggest ways to manipulate the response to mycobacteria and to improve adjuvants that stimulate the immune system.

## Introduction

**FIGURE 1.**
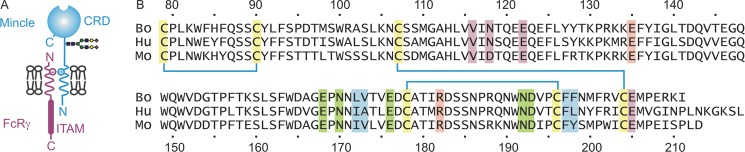
**Organization of mincle.** *A*, diagram of mincle showing the location of the CRD and association with the immunoreceptor tyrosine activation motif (*ITAM*) in the Fc receptor-γ (*FcR*γ) subunit through a charge-charge interaction in the membrane. *B*, comparison of the sequences of the predicted CRDs of bovine (*Bo*), human (*Hu*), and mouse (*Mo*) mincle. Conserved cysteine residues that form the three disulfide bonds characteristic of C-type CRDs are highlighted in *yellow*, and ligands for the conserved Ca^2+^ site are highlighted in *green* (7). Key residues in the secondary glucose-binding site are shaded *pink*, and residues that form the hydrophobic groove are shaded *blue*. Residues that chelate the supplemental Ca^2+^ are indicated in *violet*. The mouse and cow CRDs show sequence identities of 71 and 76% to the human protein, respectively.

The balance between latent and active forms of tuberculosis lies in a complex set of interactions between mycobacteria and the host immune system (1–3). In addition to tuberculosis in humans, caused by the infectious agent *Mycobacterium tuberculosis*, a bovine form of tuberculosis resulting from infection with *Mycobacterium bovis* is of wide interest because of the agricultural impact of persistent infections in domestic herds of cattle (4). Macrophages play a key role in sequestering the mycobacteria in granulomas, leading to persistent infections (2). Recent studies have revealed that the macrophage-inducible C-type lectin CLEC4E, called mincle, is an important component of communication between infecting mycobacteria and macrophages. Mincle was initially identified as a receptor in stimulated macrophages that induces cytokine release (5). It is a type II transmembrane protein, with a C-type carbohydrate recognition domain (CRD)^2^ at the C-terminal end of the extracellular domain (see Fig. 1*A*). Although the CRD contains all of the amino acid residues commonly required for sugar binding by C-type CRDs (6, 7), ligands for mincle have only recently been described. The major sugar-containing ligands are trehalose dimycolate, a glycolipid found in the outer membrane of mycobacteria (8, 9), and other mannose- or glucose-containing glycoconjugates in fungi and yeast (10, 11).

Trehalose dimycolate, which is a characteristic component of the mycobacterial surface, is also referred to as cord factor. It comprises a Glcα1–1Glcα headgroup and two complex branched and hydroxylated acyl chains attached to the 6-OH groups of each of the sugar residues. The importance of mincle in the interaction of macrophages with mycobacteria stems both from its ability to interact with trehalose dimycolate and from its association with the common Fc receptor-1γ subunit, which activates the Syk-CARD signaling pathway (12, 13). This interaction leads to granuloma formation and may participate in directing the macrophages to tolerate rather than destroy internalized mycobacteria (14).

The studies described here define the mechanism by which bovine mincle targets trehalose dimycolate. X-ray crystallography and mutagenesis studies have revealed an extended ligand-binding site in mincle that interacts with both the sugar headgroup and acyl portions of the glycolipid. Small molecule mimics of trehalose dimycolate have been generated to establish the importance of different parts of the glycolipid for recognition.

## EXPERIMENTAL PROCEDURES

### 

#### 

##### Protein Expression and Purification

Fragments of the cDNA for bovine mincle were amplified from a liver cDNA library (US Biological) by PCR (Advantage 2 Polymerase Mix, Takara), cloned into the pCR2.1-TOPO vector (Invitrogen), and sequenced using an Applied Biosystems 310 genetic analyzer. The form cloned in this way encoded a threonine residue at position 174, compared with isoleucine in the reference sequence (NCBI accession number XP_002687869), reflecting a polymorphism that has been documented in the bovine genome as rs135158086. Restriction fragments were moved into the pT5T expression vector (15) in *Escherichia coli* strain BL21(DE3). For expression of biotin-tagged proteins, nucleotides encoding the biotinylation sequence Gly-Leu-Asn-Asp-Ile-Phe-Glu-Ala-Gln-Lys-Ile-Glu-Trp-His-Glu were appended at the 3′-end of the coding sequence by inclusion in the PCR primers, and the bacteria used for expression also contained the pBirA plasmid encoding biotin ligase (16). Mutagenesis was performed by two-step PCR (17) using the original cDNA clone as template.

The wild-type CRD from cow mincle was expressed, and inclusion bodies were isolated as described previously for other C-type CRDs (18). Inclusion bodies from 6 liters of bacterial culture were dissolved in 100 ml of 6 m guanidine HCl containing 100 mm Tris-Cl (pH 7.8) and incubated in the presence of 0.01% (v/v) 2-mercaptoethanol for 30 min at 4 °C. Following centrifugation for 30 min at 100,000 × *g* in a Beckman Ti50.2 rotor, the supernatant was dialyzed against 3 changes of loading buffer containing 0.5 m NaCl, 25 mm Tris-Cl (pH 7.8), and 25 mm CaCl_2_ at 4 °C. Insoluble material was removed by centrifugation as described above, and the supernatant was applied to a 10-ml column of trehalose-Sepharose prepared by coupling with divinyl sulfone (19). After rinsing with 14 ml of 150 mm NaCl, 25 mm Tris-Cl (pH 7.8), and 25 mm CaCl_2_, the bound protein was eluted with 16 aliquots of 1 ml of 150 mm NaCl, 25 mm Tris-Cl (pH 7.8), and 2.5 mm EDTA. Fractions containing the CRD were determined by examining aliquots on SDS-polyacrylamide gels containing 17.5% (w/v) acrylamide, which were stained with Coomassie Blue. The purified protein was characterized by gel filtration on a 1 × 30-cm Superdex 200 column (GE Healthcare) eluted with 10 mm Tris-Cl (pH 7.8), 100 mm NaCl, and 2.5 mm EDTA at a flow rate of 0.5 ml/min, with absorbance monitored at 280 nm. Mutant proteins were purified by the same protocol, except that invertase-Sepharose was used in place of trehalose-Sepharose.

##### Binding Assays

Glycolipid blotting experiments were performed by spotting 2–4 μl of the following glycolipid solutions on polyvinylidene difluoride membrane (Millipore): trehalose dimycolate (Carbosynth Ltd.) at 2 μg/μl in chloroform, galactosylceramide from bovine brain (Sigma) at 2 μg/μl in chloroform/methanol (2:1), and lipoteichoic acid from *Staphylococcus aureus* (Sigma) at 2 μg/μl in water/methanol (2:3). After the spots were dry, the membrane was wetted with methanol, immersed immediately in a solution of 5% (w/v) BSA in binding buffer (1.5 m NaCl, 25 mm Tris-Cl (pH 7.8), and 2.5 mm CaCl_2_), and incubated for 1 h at room temperature with shaking. The biotin-tagged CRD from bovine mincle was added to a final concentration of 1 μg/ml and incubated for an additional 1.5 h at room temperature. Following three washes of 5 min each with binding buffer, the membrane was incubated with 0.5 μg/ml ExtrAvidin coupled to alkaline phosphatase (Sigma) in 5% (w/v) BSA in binding buffer for 1 h at room temperature. After an additional three washes with binding buffer, the blot was developed with nitro blue tetrazolium and 5-bromo-4-chloro-3′-indolyl phosphate substrate (Millipore).

Immobilized CRDs for competition binding assays were prepared in streptavidin-coated wells (Thermo Scientific Pierce), washed three times with binding buffer, incubated with biotin-tagged CRDs at 5 μg/ml in 0.1% (w/v) BSA in binding buffer for 2 h at 4 °C, and washed an additional three times with binding buffer. Competing ligands were incubated in the wells in the presence of 0.5 μg/ml ^125^I-Man_31_-BSA in 0.1% (w/v) BSA in binding buffer for 2 h at 4 °C. After washing three times with binding buffer, wells were counted in a Wallac WIZARD gamma counter (PerkinElmer Life Sciences). Assays were performed in duplicate, with the average values used to calculate inhibition constants using SigmaPlot for fitting to a simple binding curve (20). The order of binding determined from the fitting was always in the range of 0.8–1.3. Ratios of *K_I_*_(trehalose)_/*K_I_*_(acyltrehalose)_ were calculated for assays performed together on one assay plate. Each set of assays was then repeated three to four times. The reported values are means ± S.D. for the ratios from replicate experiments. The pH dependence of ligand binding was measured in the same assay format, with no competing ligand and with a CaCl_2_ concentration of 2.5 mm. A series of buffers, each containing both 25 mm MES and 25 mm MOPS and adjusted to various pH values with NaOH, were substituted for the Tris buffer.

##### Crystallization and Data Collection

The untagged CRD was concentrated by extensive dialysis against water and lyophilization before being dissolved in 2.5 mm CaCl_2_, 10 mm Tris-Cl (pH 8.0), and 25 mm NaCl. Crystals were obtained by hanging-drop vapor diffusion using a mixture of 3:1 and 2:1 μl of protein/reservoir in the drops for the unliganded and liganded forms, respectively. Crystals were grown at 22 °C from a protein solution comprising 2.2–3.5 mg/ml mincle, 2.5 mm CaCl_2_, 10 mm Tris-Cl (pH 8.0), and 25 mm NaCl. Trehalose (50 mm) was added to the protein solution to form the complex. The protein/trehalose solution precipitated slightly but cleared while forming the crystallization drop. The reservoir solution used to obtain crystals of the native protein contained 20% (w/v) polyethylene glycol 4000, 20% (v/v) 2-propanol, and 0.1 m sodium citrate (pH 5.0), and the reservoir solution for the trehalose complex contained 25% (w/v) polyethylene glycol 4000, 17.5% (v/v) 2-propanol, and 0.1 m sodium citrate (pH 5.0). Crystals were transferred to perfluoropolyether (PFO-XR75, Lancaster Synthesis) before being frozen in liquid nitrogen for data collection. Diffraction data were measured at 100 K at the Stanford Synchrotron Radiation Lightsource. Data were processed with XDS and scaled with SCALA (21). Data collection statistics are summarized in Table 1.

##### Structure Determination

The structure of the native mincle CRD was solved by molecular replacement using the program Phaser (22). The model used for molecular replacement was derived from Protein Data Bank entry 1SL4, removing the saccharide, Ca^2+^, water molecules, and residues 253–255 and 283–284. The molecular replacement solution confirmed the existence of two monomers, designated A and B, in the asymmetric unit and showed that the crystals belong to space group *P*6_5_. Model building and refinement were performed with Coot (23) and PHENIX (24). Refinement included individual positional isotropic temperature factor and TLS refinement. A difference Fourier map showed strong peaks at two positions in each monomer, one at the expected primary Ca^2+^ site and another Ca^2+^ at a site found in some other C-type lectins, as well as a citrate molecule bound at the primary Ca^2+^ site of monomer A. Refinement of models with either Ca^2+^ or Na^+^ revealed that Ca^2+^ is present in these sites, as indicated by the comparable temperature factors of Ca^2+^ and its surrounding atoms *versus* a very low refined value for Na^+^. The presence of Ca^2+^ was confirmed by the presence of peaks in an anomalous difference Fourier map (Δ*f*″ = 0.57 *e* for Ca^2+^ *versus* 0.05 *e* for Na^+^ at 12.6 keV). In the trehalose complex, these peaks are at heights of 25 and 25 σ in copy A and of 19 and 6 σ in the less well defined copy B. In the native crystal, the peaks are at 6 and 9 σ in copy A and at 3 and 7 σ in copy B. In contrast, electron density modeled as Na^+^ showed no significant peaks in the anomalous difference Fourier map.

The structure of the trehalose complex was solved by molecular replacement using monomer A of a partially refined structure of the native CRD. The solution confirmed space group *P*4_1_2_1_2, with two monomers in the asymmetric unit. Anisotropic temperature factors were refined in monomer A in this structure. The atomic coordinates and structure factors have been deposited in the Protein Data Bank, with code 4KZW for the native CRD and code 4KZV for the trehalose complex.

##### Synthesis of Trehalose Derivatives

Reagents for synthesis were obtained from Sigma, and chromatography media were from Merck. Acylated derivatives of trehalose were prepared by reacting 500 mg of trehalose with 1–5 ml of carboxylic acid in the presence of 500 mg of lipase from *Candida antarctica* immobilized on polystyrene beads in 25 ml of isoamyl alcohol at 40 °C with mixing (25). The lower concentration of acid was used to favor synthesis of the monoacylated derivatives. Mono- and diacylated derivatives were separated by chromatography on 25 ml of silica gel in chloroform/methanol/water (75:25:4). Fractions of 5 ml were collected. Purification was monitored by thin layer chromatography on aluminum-backed silica gel plates developed in the same solvent and stained with orcinol/sulfuric acid reagent (supplemental Fig. S1) (26). Purified compounds were dried from solvent, dissolved in water, filtered through Anotop 20-nm pore filters (Whatman), and lyophilized. Stock solutions were prepared in water and assayed using the anthrone reaction (27). Compounds were characterized by matrix-assisted laser desorption ionization mass spectrometry on an Applied Biosystems 4800 instrument and by one-dimensional proton NMR on a Bruker 400-MHz spectrometer (supplemental Figs. S2 and S3).

## RESULTS

### 

#### 

##### Characterization of the Mincle CRD

**FIGURE 2.**
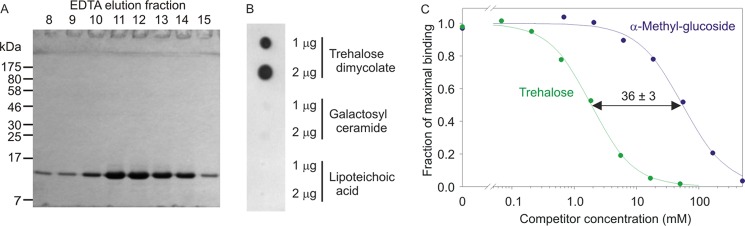
**Binding specificity of mincle.** *A*, SDS-PAGE of the CRD from bovine mincle expressed in *E. coli* and purified on trehalose-Sepharose. Refolded protein, which bound to the affinity column in the presence of Ca^2+^, was eluted with EDTA. The gel was stained with Coomassie Blue. *B*, glycolipid blot demonstrating bovine mincle binding to trehalose dimycolate. The blot was incubated with the biotin-tagged CRD from bovine mincle, followed by alkaline phosphate-conjugated streptavidin. *C*, quantitative comparison of the affinities of trehalose and glucose for mincle determined in a binding competition assay in which the biotin-tagged CRD immobilized on streptavidin-coated wells was probed with ^125^I-Man_31_-BSA. An example of one assay, performed in duplicate, is shown. The mean ratio of *K_I_* values for α-methyl glucoside and trehalose is based on four repetitions of the assay.

Comparison of mouse, human, and cow mincle reveals close sequence similarity throughout the polypeptides, including in the C-terminal CRD (Fig. 1*B*). A minimal CRD from bovine mincle was expressed in *E. coli* and could be purified by affinity chromatography on immobilized trehalose (Fig. 2*A*). Binding of the mincle CRD to trehalose dimycolate was demonstrated using a glycolipid overlay assay (Fig. 2*B*). Competition assays using the unusual 1–1′-linked disaccharide trehalose headgroup of the glycolipid revealed a 36-fold higher affinity for the disaccharide compared with the constituent glucose monosaccharides (Fig. 2*C*), indicating the presence of an extended binding site encompassing both glucose residues.

**FIGURE 3.**
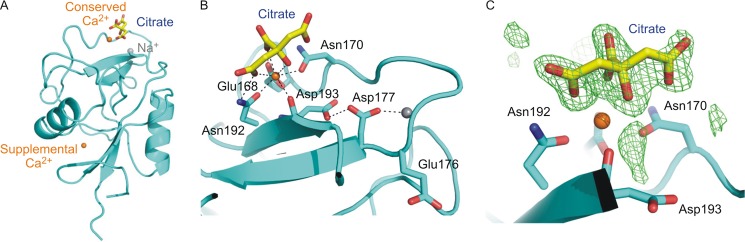
**Structure of a low pH form of the mincle CRD.** *A*, overall structure of the mincle CRD in crystals obtained at pH 5.0, in which citrate is bound at the conserved Ca^2+^ site. Ca^2+^ is shown in *orange*, and Na^+^ is shown in *gray*. The supplemental Ca^2+^-binding site, seen near the bottom of the structure, has been observed in other C-type CRDs (40). *B*, region of the CRD around the conserved Ca^2+^ site at which the sugar-binding site is usually located, showing a bound citrate ion. The side chain of one of the expected ligands, Asp-193, is rotated away from the Ca^2+^, and a second expected ligand, Glu-176, is in a loop encompassing Asn-170–Asp-177, which assumes an unusual conformation in which Glu-176 does not contact the conserved Ca^2+^ site. Hydrogen and coordination bonds are indicated with *dotted lines*, using cutoffs of 2.6 Å for coordination bonds and 3.2 Å for hydrogen bonds. *C*, *F_o_* − *F_c_* electron density for the citrate moiety, calculated by omitting the citrate from the model, contoured at 3.0 σ, and shown as *green mesh*. Oxygen atoms are indicated in *red*, and nitrogen atoms are *blue*.

Initial crystals of the expressed CRD, obtained in citrate buffer at pH 5, were solved by molecular replacement with a search model based on the CRD of DC-SIGN (Protein Data Bank entry 1SL4). The data statistics are summarized in Table 1, and the refinement statistics are shown in Table 2. The mincle polypeptide assumes an overall fold similar to other C-type CRDs (Fig. 3*A*) (7). As in these other structures, the polypeptide assumes a loop-out topology, in which β-strands at the N and C termini are paired at the bottom of the structure as it is presented in Fig. 3*A* and are flanked by two helices. The upper portion of the structure consists largely of a series of irregular loops surrounding a short pair of β-strands. However, in the conserved Ca^2+^-binding site that serves as the primary sugar-binding site in C-type lectins (7), only three of the five amino acid side chains that normally form the divalent cation-binding site (Fig. 1*B*) are coordinated to the Ca^2+^ (Fig. 3*B*). Despite these differences, a Ca^2+^ is found at the conserved site, coordinated to three oxygens of a citrate molecule (Fig. 3, *B* and *C*).

**TABLE 1:** **Crystallographic data statistics**

Data	Native mincle CRD	Trehalose mincle CRD
SSRL*^a^* beamline	11-1	12-2
Wavelength (Å)	0.97945	0.97950
Space group	*P*6_5_	*P*4_1_2_1_2
Unit cell lengths (Å)	*a* = *b* = 64.29, *c* = 127.16	*a* = *b* = 73.48, *c* = 99.3
Resolution (Å)	33.72–1.85 (1.95–1.85)*^b^*	36.74–1.40 (1.48–1.40)
*R*_sym_*^c^*	0.038 (0.42)	0.034 (0.34)
CC_*I*mean	1.00 (0.96)	1.00 (0.96)
Mean ((*I*)/σ(*I*))	33.4 (6.0)	29.6 (6.3)
Completeness (%)	98.2 (97.8)	99.8 (99.0)
No. of unique reflections	24,890	54,041
Average multiplicity	10.4 (10.6)	9.6 (9.7)

*^a^* SSRL, Stanford Synchrotron Radiation Lightsource.

*^b^* Values in parentheses are for the highest resolution shell.

*^c^ R*_sym_ = Σ*_h_*Σ*_i_*(|*I_i_*(*h*)| − |〈*I*(*h*)〉|)/Σ*_h_*Σ*_i_I_i_*(*h*), where *I_i_*(*h*) is the observed intensity, and 〈*I*(*h*)〉 is the mean intensity obtained from multiple measurements.

**TABLE 2:** **Crystallographic refinement statistics**

Data	Native mincle CRD	Trehalose mincle CRD
**Residues in final model***^a^*		
Copy A		
Protein residues	79–211	78–208
Ca^2+^	2	2
Ligand at primary Ca^2+^ site	Citrate	Trehalose
Copy B		
Protein residues	78–171, 175–211	78–171, 177–209
Ca^2+^	2	2
Water molecules	154	273

**Reflections used for refinement**	23,643	51,275

**Reflections marked for *R*_free_**	1245	2694

***R*_free_*^b^***	22.5	20.2

***R*_cryst_*^b^***	17.9	16.5

**Average *B* factor**	48.2	29.3

**Bond length r.m.s.d. (Å)*^c^***	0.010	0.010

**Angle r.m.s.d.**	1.0°	0.97°

**Ramachandran plot (%)*^d^***		
Preferred	93.0	92.8
Allowed	5.1	7.2
Outliers	1.9	0.0

*^a^* Construct contains residues 78–211.

*^b^ R* and *R*_free_ = Σ‖*F_o_*| − |*F_c_*‖/Σ|*F_o_*|, where |*F_o_*| is the observed structure factor amplitude, and |*F_c_*| is the calculated structure factor amplitude for the working and test sets, respectively.

*^c^* r.m.s.d., root mean square deviation.

*^d^* As defined in Coot.

##### Structure of the Mincle CRD Bound to Trehalose

**FIGURE 4.**
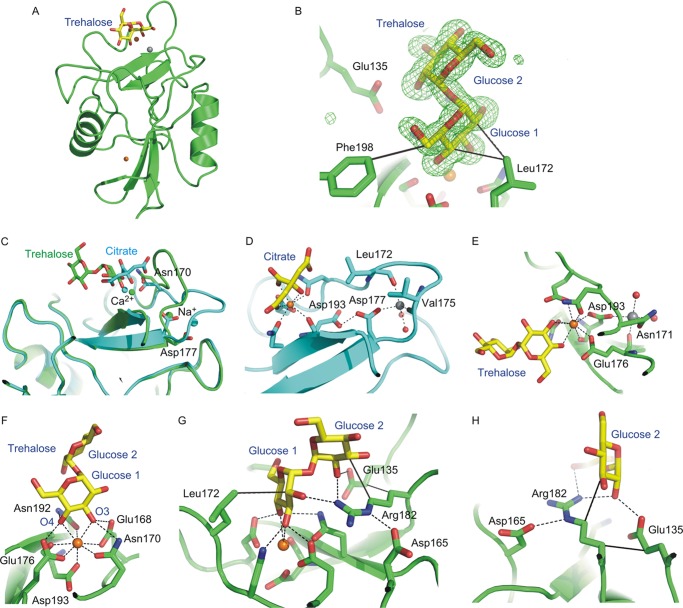
**Structure of mincle complexed with trehalose.** *A*, overall structure of the trehalose-CRD complex. *B*, *F_o_* − *F_c_* electron density for the trehalose saccharide, calculated by omitting the sugar residue from the model, contoured at 3.0 σ, and shown as *green mesh. C*, superposition of the mincle CRD bound to citrate (*blue*) and complexed with trehalose (*green*), showing the different conformation of the loop between Asn-170 and Asp-177 near the conserved Ca^2+^ site. *D*, arrangement of cation-binding sites in the citrate complex. A Na^+^ (*gray sphere*) interacts with the main chain oxygens of Leu-172 and Val-175, as well as with the side chain of Asp-177 and two water molecules. *E*, arrangement of cation-binding sites in the trehalose complex. In this case, the Na^+^ interacts with the main chain oxygen of Glu-176 and the carboxyl group of Asp-193, as well as the side chain of Asn-171 and two water molecules. *F*, primary binding site for glucose at the conserved Ca^2+^ site showing the five canonical ligands for the divalent cation, four of which also interact with the 3- and 4-OH groups of the first glucose residue. *G*, trehalose disaccharide interactions with both the primary and secondary binding sites. *H*, secondary binding site, in which Glu-135 and Arg-182 form hydrogen bonds with 2-OH of the second glucose residue. Ca^2+^ is shown in *orange*, Na^+^ in *gray*, oxygen atoms in *red*, and nitrogen atoms in *blue*. Hydrogen and coordination bonds are indicated with *dashed lines*, using cutoffs of 2.6 Å for coordination bonds and 3.2 Å for hydrogen bonds. van der Waals contacts, based on a 4.0-Å cutoff, are indicated with *solid lines*.

Because Ca^2+^ and sugar binding are coupled in C-type lectins (28), a high carbohydrate ligand concentration should shift the conformational equilibrium of the conserved Ca^2+^ site toward the sugar-binding conformation. Generating the mincle CRD crystals in the presence of trehalose resulted in a remarkable change in the organization of the crystals, from space group *P*6_5_ to *P*4_1_2_1_2, and the diffraction limit for the crystals improved from 1.85 to 1.4 Å. Solving the structure of this new crystal form using the original structure as a search model revealed the presence of a trehalose molecule bound to the CRD (Fig. 4, *A* and *B*). There are substantial rearrangements of the polypeptide in the trehalose-bound structure (Fig. 4, *C–E*). The side chains of Asp-193 and Glu-176 shift into the positions observed in other C-type CRD Ca^2+^-binding sites such that the trehalose complex has the expected arrangement in which five side chains are now ligands for the conserved Ca^2+^ site (Fig. 4*C*).

In both the native and trehalose-bound structures, a Na^+^ ion is present near the Ca^2+^ (Fig. 4, *D* and *E*), but it interacts with different residues, shifting closer to the Ca^2+^ in the trehalose complex so that both Asp-193 and Glu-176 bridge between the Ca^2+^ and Na^+^. This region appears to be flexible, as it is poorly ordered in copy B of both the native and trehalose-bound structures. The change in space group is likely due to the different ordered conformations of this region present in copy A, which is packed in different lattice environments. Given that the native and trehalose-bound crystals were obtained under virtually identical conditions, the observed differences in conformation (and therefore, lattice packing) can be attributed to the presence of saturating trehalose. However, it is possible that the apparent flexibility is a consequence of the low pH.

**FIGURE 5.**
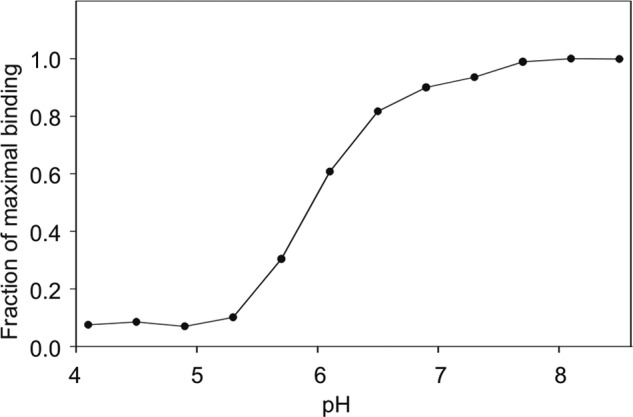
**pH dependence of ligand binding to mincle CRD.** The binding of ^125^I-Man_31_-BSA to the biotin-tagged CRD immobilized on streptavidin-coated wells was measured.

The unusual conformation of the conserved Ca^2+^ site in the native crystals compared with the trehalose complex may be related to the low pH crystallization condition, which is predicted to disrupt interactions of acidic side chains with Ca^2+^. Because sugar binding depends on the precise arrangement of ligands in the Ca^2+^ site, loss of sugar-binding activity at low pH, resulting from reduced affinity for Ca^2+^, is characteristic of C-type CRDs that release their ligands in endosomes following endocytosis (29, 30). As predicted, the affinity for ligand was dramatically diminished at low pH (Fig. 5).

One of the two glucose residues in the bound trehalose molecule occupies the canonical primary binding site typical of C-type CRDs, with the equatorial 3- and 4-OH groups forming coordination bonds to the Ca^2+^ and hydrogen bonds with four of the amino acid side chains that also ligate the Ca^2+^: Glu-176, Asn-192, Glu-168, and Asn-170 (Fig. 4*F*). This arrangement of the equatorial 3- and 4-OH groups and side chains mimics the coordination of mannose and *N*-acetylglucosamine residues in DC-SIGN, mannose-binding protein, and other C-type CRD-ligand complexes (6, 7, 31). The A face of the glucose is packed against the side chain of Leu-172 (Fig. 4*G*), which prevents an alternative arrangement in which the positions of 3- and 4-OH groups are switched by a rotation of 180° because the presence of the second glucose residue would clash with the leucine side chain.

Trehalose bound to mincle falls in the ensemble of conformations observed in other crystal structures of unrelated proteins (for example, Protein Data Bank entry 1EU8, trehalose/maltose-binding protein from *Thermococcus litoralis*; entry 1F0P, *M. tuberculosis* antigen 85B; entry 2DXY, C-terminal lobe of bovine lactoferrin; and entry 3OIH, xylanase-α-amylase inhibitor protein) and in solution (32). The arrangement of the two glucose residues relative to each other brings the second glucose residue into contact with additional portions of the surface of the protein, which form a secondary binding site (Fig. 4, *G* and *H*). In this site, glucose 2-OH is part of a cooperative hydrogen bonding network in which it accepts a hydrogen bond from Arg-182 and donates to Glu-135, thereby bridging these two side chains. Glucose C3 also packs against the Arg-182 side chain, which in turn also packs against Glu-135 (Fig. 4, *G* and *H*).

**FIGURE 6.**
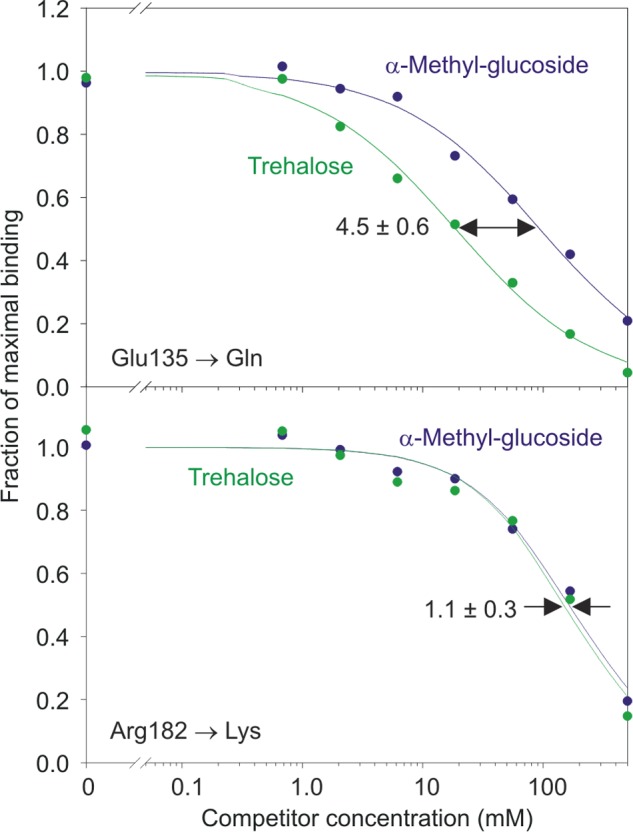
**Mutagenesis of residues in the secondary glucose-binding site in mincle.** An example of one assay, performed in duplicate, is shown. The ratios of the *K_I_* values for α-methyl glucoside and trehalose following mutation of Glu-135 to glutamine and mutation of Arg-182 to lysine are reported as means based on four repetitions of the assay.

Changing Glu-135 to glutamine, reducing its effectiveness as a hydrogen bond acceptor, resulted in an 8-fold reduction of the enhanced binding of trehalose compared with glucose (Fig. 6). Changing Arg-182 to lysine reduced the affinity for trehalose to being the same as that for α-methyl glucoside, suggesting that the lysine side chain cannot form hydrogen bonds with 2-OH of the second glucose, thus effectively destroying the secondary binding site. The importance of Arg-182 also reflects the fact that it forms a hydrogen bond with 2-OH of the glucose in the primary binding site and packs against C3 of the glucose residue in the secondary binding site (Fig. 4, *G* and *H*). These results confirm that the secondary sugar-binding site observed in the crystal structure could function as part of the binding mechanism for trehalose dimycolate.

##### Binding of Glycolipid Analogs to the Mincle CRD

**FIGURE 7.**
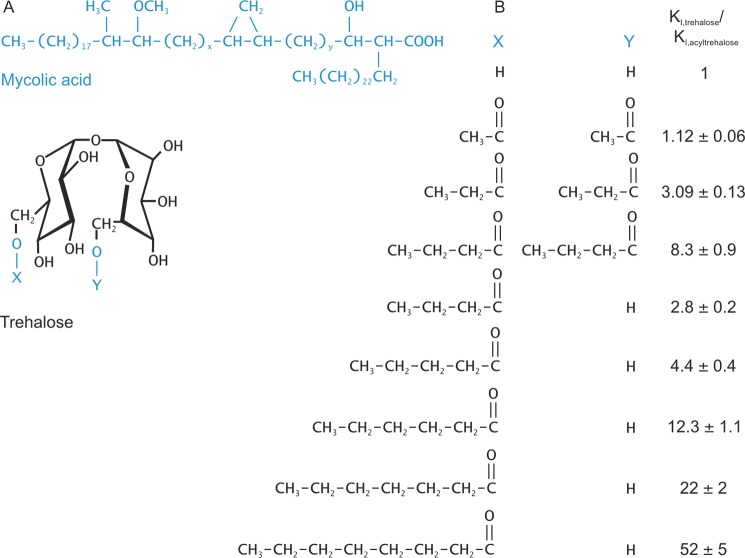
**Mincle interactions with glycolipid mimics.** *A*, structures of mycolic acid and trehalose. Both *X* and *Y* are mycolic acid in trehalose dimycolate. *B*, enhancement of the affinity of mincle for trehalose by acylation of one or both of the 6-OH groups demonstrated in binding competition assays. Values are means ± S.D. for three to four replicate experiments.

In the predominant mycobacterial glycolipid, both of the 6-OH groups of the glucose residues in trehalose are esterified with the unusual α-branched, β-hydroxylated mycolic acid (Fig. 7*A*). As a soluble mimic of this structure, trehalose derivatives esterified on the 6-OH groups with simple linear acids were prepared by enzymatic synthesis using immobilized lipase under anhydrous conditions to achieve regioselective modification of the primary hydroxyl (6-OH) groups (25). Binding competition assays demonstrated that acetylation at the two 6-OH groups is tolerated, confirming that these groups are unencumbered on the surface of the CRD. In addition, acylation with 3- or 4-carbon chains resulted in significantly enhanced affinities for mincle, reflected in an increase in the *K_I_*_(trehalose)_/*K_I_*_(acyltrehalose)_ values (Fig. 7*B*).

**FIGURE 8.**
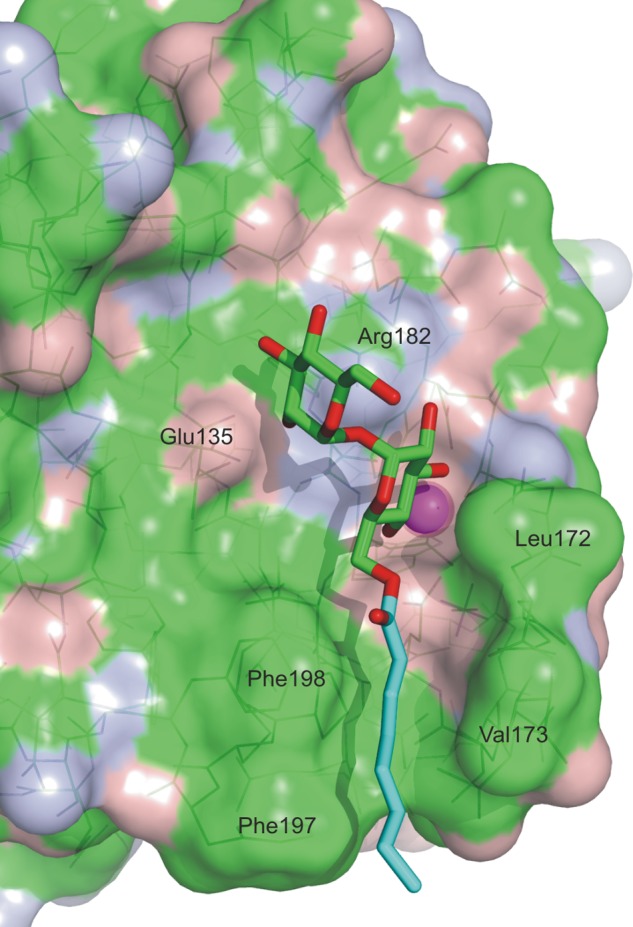
**Hydrophobic channel adjacent to the primary binding site.** A model of the trehalose octanoate conjugate was made by adjusting the O5-C5-C6-O6 dihedral angle of glucose residue 1 by 120° and forming a bond of the appropriate length with the planar carboxylate group of octanoic acid from Protein Data Base entry 1H2B. The model suggests that at least the first six carbon atoms of the acyl chain would interact with the channel. The surface of the protein is colored based on the underlying atoms: *green* for carbon, *red* for oxygen, and *blue* for nitrogen. The hydrocarbon chain of the octanoic acid is colored *cyan*.

Examination of the surface of the mincle CRD near the primary sugar-binding site reveals a hydrophobic channel running between Phe-197/Phe-198 on one side and Leu-172/Val-173 on the other side (Fig. 8). The top of this channel is directly adjacent to the 6-OH group of the glucose residue in the primary binding site. Modeling of octanoic acid attached to this 6-OH group suggests that at least six carbon atoms would be accommodated within the channel (Fig. 8). Because the 6-OH group of the other glucose residue is positioned away from the surface of the CRD, ligands in which a single fatty acid chain was attached to one of the two glucose residues were synthesized to maintain solubility of the ligands. As expected, the mono-*n*-butanoyl derivative showed enhanced affinity compared with trehalose, and increasing the length of the fatty acid from four to eight carbons resulted in further increases in affinity (Fig. 7*B*). The fatty acid chains alone did not compete for binding at concentrations up to 50 mm, which is 20–1000-fold higher than the *K_I_* values of the acylated trehalose derivatives (data not shown).

**FIGURE 9.**
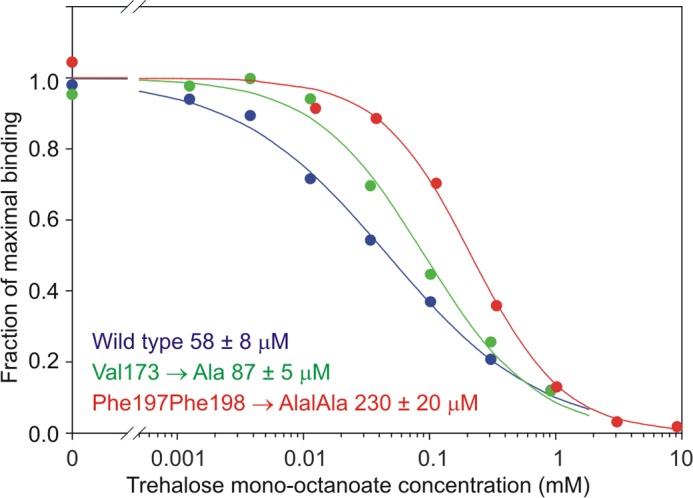
**Mutagenesis of residues in the hydrophobic groove in mincle.** Inhibition curves for the mono-octanoyl derivative of trehalose competing for binding to wild-type and mutant mincle CRDs are shown. *K_I_* values for trehalose mono-octanoate (based on four repetitions of the assay) are indicated.

The model suggests that much of the increase in affinity for the acylated trehalose derivatives may result from favorable interactions with the hydrophobic groove, although some contribution of decreased solubility of the longer chain ligands cannot be ruled out. To obtain direct evidence for interaction of the acyl chains with the protein surface, mutations were made in the amino acid residues that form the hydrophobic groove. Removal of one side of the channel by mutation of Phe-197 and Phe-198 resulted in a 4-fold loss in affinity for mono-octanoyl trehalose based on the binding competition assay (Fig. 9). The other side of the groove could only be partially removed because Leu-172 forms part of the primary glucose-binding site. However, mutation of Val-173 resulted in a 1.5-fold reduction in affinity for the acylated ligand. These results are consistent with the proposed mode of acyl chain binding shown in Fig. 8.

## DISCUSSION

**FIGURE 10.**
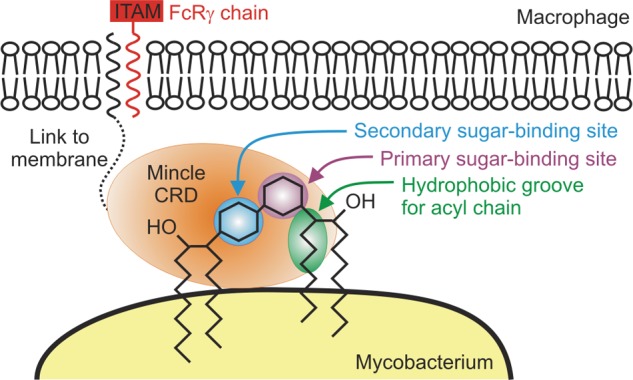
**Model of the interaction of mincle with mycobacteria.** Mincle, on the surface of macrophages, is shown forming a bridge with trehalose dimycolate on the surface of mycobacteria. *ITAM*, immunoreceptor tyrosine activation motif; *FcR*γ, Fc receptor-γ subunit.

The structural and binding data presented here support a model for binding of trehalose dimycolate in which one of the acyl chains of the lipid interacts directly with the CRD, providing specificity directed by both the sugar and lipid portions of the mycobacterial ligand (Fig. 10). The structural and biochemical description of the ligand-binding site in mincle presented here provides a novel paradigm for interaction of a glycan-binding receptor with a glycolipid target. The binding site consists of a canonical C-type primary binding site centered on Ca^2+^, supplemented on one side by a secondary binding site for the second glucose residue in the trehalose headgroup and on the other side by a hydrophobic channel that can bind acyl groups. In combination, these three sites provide an ideal mechanism for recognition of the unique features of a pathogen-specific glycolipid. The glucose diglycoside is essentially clamped between the primary and secondary sugar-binding sites, with stereospecificity derived from a network of hydrogen bonds to hydroxyl groups on each sugar, as well as non-polar contacts. In addition to allowing hydrophobic interactions with the linear hydrocarbon portion of mycolic acid, the open side of the hydrophobic channel accommodates the branching and hydroxylation of this mycobacterium-specific surface structure. The interaction of both the polar and non-polar portions of trehalose dimycolate with adjacent sites on the mincle CRD contrasts with the T-cell receptor-CD1 complex, in which the headgroup and acyl portions of a glycolipid are bound by separate receptors on two different cells (33).

C-type lectins (such as DC-SIGN) that are involved in pathogen recognition in innate immunity show high degrees of structural and functional divergence between species (34). In contrast, sequence conservation (Fig. 1*B*) suggests that the trehalose dimycolate-binding site described here is conserved in human and mouse orthologs of bovine mincle. Conservation of the mechanism for interaction with trehalose dimycolate across mammalian species may reflect evolutionary coexistence of mammalian hosts and mycobacterial pathogens (35). The arrangement of a hydrophobic channel adjacent to the sugar-binding site in mincle suggests how other glycolipids with alternative headgroups, such as those found in the fungus *Malassezia* (10), might bind to and activate mincle. On the other hand, the absence of the residues that form the hydrophobic channel from the related macrophage receptor C-type lectin (MCL) indicates that weak binding of this additional receptor to trehalose dimycolate (36) probably involves the headgroup only.

Formation of the hydrophobic groove depends on the loop between residues 170 and 177 assuming the conformation observed in the trehalose complex, so the different conformation of this region observed in the native crystals may contribute to the observed pH dependence of ligand binding. The finding that binding would be reversed under endosomal conditions in turn provides a mechanism for down-regulation of signaling following internalization of mycobacteria. Interaction of mincle with a portion of the acyl chains attached to trehalose may also result in disruption of the mycobacterial membrane organization.

The action of trehalose dimycolate or cord factor as an adjuvant has been widely exploited (37–39). In addition to yielding insights into mycobacterial interactions with host macrophages, this investigation reveals how ligands such as the synthetic adjuvant trehalose dibehenate interact with the CRD in mincle and provides a basis for further development of adjuvants.

## Footnotes

2 The abbreviation used is:

CRD: carbohydrate recognition domain.
